# The influence of yeast level and fermentation temperature on Ochratoxin A decrement during bread making

**DOI:** 10.1002/fsn3.1059

**Published:** 2019-05-07

**Authors:** Payman Mozaffary, Jafar M. Milani, Ali Heshmati

**Affiliations:** ^1^ Department of Food Science and Technology Sari Agricultural Sciences and Natural Resources University Mazandaran Iran; ^2^ Department of Nutrition and Food Safety, School of Medicine Nutrition Health Research Center, Hamadan University of Medical Sciences Hamadan Iran

**Keywords:** bread, fermentation temperature, Ochratoxin A, *Saccharomyces cerevisiae*, yeast level

## Abstract

Ochratoxin A (OTA) occurrence in cereals is a permanent challenge in human health. In recent years, some studies have focused on the role of yeasts as adsorbing tools to eliminate OTA. The aim of the current research was to study the effects of different variables including *Saccharomyces cerevisiae* amount and the fermentation temperature on the reduction of OTA during bread baking. For this purpose, the OTA was spiked to the flour and then the bread was prepared. OTA levels in flour, dough, and bread were measured by the high‐performance liquid chromatography with fluorescence detector (HPLC‐FD). The results revealed that yeast level and fermentation temperature had a significant effect on OTA reduction. The increase of the amount of *S. cerevisiae* from 1% to 2% w/w and the fermentation temperature from 25ºC to 30ºC resulted in the increment of OTA reduction from 31.17% to 59.41%. During baking, OTA reduction was 19.21%. In general, the utilization of 2% w/w *S. cerevisiae* and the fermentation temperature of 35 ºC could reduce considerable amount of OTA in the wheat bread.

## 1 INTRODUCTION

Cereal products, such as wheat and rice, are particularly important for humans due to their role as main food crops in the various areas of the world (Hasani, Khodadadi, & Heshmati, 2016; Ranjbar, Heshmati, Momtaz, & Vahidinia, 2019). One of the most challenging concerns in the preservation and the storage of crops, particularly grains, is their contamination by mycotoxins (Duarte, Pena, & Lino, 2010). Human exposure to different mycotoxins increases health concerns (Mashak, Sohi, Heshmati, & Nejad, 2016). Mycotoxins are known as extracellular‐secreted secondary metabolites produced by various fungi species, such as *Aspergillus, Penicillium,* and *Fusarium* (Heshmati, Ghadimi, Ranjbar, & Khaneghah, 2019). These fungi can grow on both food and feed under different conditions of temperature and humidity, with diverse toxic effects. Indeed, approximately 25% of cereals consumed in the world are contaminated by mycotoxins (Akpo‐Djènontin, Gbaguidi, Soumanou, & Anihouvi, 2018)**.**

Ochratoxin A (OTA) is the secondary metabolite of *Aspergillus* strains and *penicillium* (Heshmati & Mozaffari Nejad, 2015; Mansouri‐Nasrabadi, Milani, & Nazari, 2018; Valle‐Algarra et al., 2009). OTA has been classified as human carcinogenic, in the Group 2B, by the IARC (IARC, 2002). Previous studies have shown that OTA is nephrotoxic mycotoxin with carcinogenic, immunotoxic, mutagenic, and neurotoxic properties (Heshmati & Mozaffari Nejad, 2015; Heshmati, Zohrevand, Khaneghah, Nejad, & Sant’Ana, 2017).

The occurrence of OTA in various products including cereals, coffee, grape juice, wine, spices, dried fruit, and raisins as well as several animal‐derived food products (meat, egg, and blood) was reported (Duarte et al., 2010; Jørgensen, 2005; Milani, 2013; Paoloni, Solfrizzo, Bibi, & Pecorelli, 2018; Sousa et al., 2019; Viegas et al., 2018) . OTA is one of the major problems of wheat, barley, and other small grains around the world, affecting the yield and the quality of grain, and the products achieved from these contaminated materials (Zhang & Wang, 2014).

The process of cereal grains could influence its OTA contents (Milani & Maleki, 2014). In previous researches, the effects of processing procedures such as cleaning, milling, extrusion, fermentation, baking, and frying on the fate of OTA have been studied. Scudamore, Banks, and MacDonald (2003) found that cleaning and dry‐milling decreased the OTA level in white flour about 55% to 75% (Scudamore et al., 2003). Furthermore, Scudamore, Banks, and Guy (2004) described that OTA was reduced by 8% to 42% during processing of wheat flour (Scudamore et al., 2004).

In addition, the thermal processes and fermentation can influence OTA. Although it seems that OTA is very stable at high temperatures and resistant to hydrolysis, some studies reported its reduction during baking (Vidal, Marín, Morales, Ramos, & Sanchis, 2014; Vidal, Morales, Sanchis, Ramos, & Marín, 2014). Dahal, Lee, Gu, and Ryu (2016) studied the reduction of OTA during various heating times (up to 60 min) at different temperatures (100, 125, 150, 175, and 200ºC) in the aqueous buffer solutions at different pH (pH 4, 7, and 10). Over 90% of OTA was reduced at 200ºC in pH 4. After processing under an alkaline condition (pH 10) at 100ºC for 60 min, about 50% of the OTA was lost (Dahal et al., 2016). Valle‐Algarra et al. (34) reported that about 30% of OTA was reduced during dough fermentation with *S. cerevisiae*, depending on the OTA levels added to the wheat flour (Valle‐Algarra et al., 2009). Milani and Heidari (2017) found the fermentation stage during bread making had the greatest effect on the OTA (Milani & Heidari, 2017).

There is little information regarding the impact of yeast level and the fermentation temperature on OTA. The aim of present study was to study the effects of different variables including various levels of *S. cerevisiae* and the fermentation temperature on the reduction of OTA during bread baking.

## 2 MATERIALS AND METHODS

### 2.1 Chemicals

Crystalline OTA acquired from Sigma was dissolved in methanol to prepare stock standard solutions with the concentration of 1,000 μg/mL and kept at −20°C in a dark glass. Acetonitrile, phosphate‐buffered saline (PBS), methanol, ethanol, and acetic acid were purchased from Merck. All the solvents were HPLC purity grade. Filter papers (Whatman No. 1) were purchased from Whatman (Whatman International Ltd). Immunoaffinity chromatography columns (IAC) for OTA cleanup were from Libios (Bully, France). Pure water was prepared using a Milli‐Q apparatus (Millipore). *S. cerevisiae* was purchased from Khamir Mayeh‐e‐Razavi. Wheat flour was purchased from the local market in Sari city, Iran.

### 2.2 Spiking OTA into flour

The European Commission established the maximum limit for OTA in the raw cereal grains and all products derived from cereals for the direct human consumption, as 5 μg/kg and 3 μg/kg, respectively (Commission, 2002). In this study, wheat flour was initially analyzed for OTA, according to which the level of OTA was below the detection limits of the methods used in this work. One milliliter of standard OTA solution (1,000 μg/L) was diluted with 9 ml of methanol and stored at 4°C in a sealed vial until use. Then, 5 ml of this solution was added to 100 g of flour to achieve the OTA contamination level of 5 µg/kg.

### 2.3 Bread making

Dough was prepared with mycotoxin‐spiked flour, water, yeast, and sodium chloride. Firstly, 100 g of OTA spiked flour was mixed with 2 g edible salt or sodium chloride (purity: 99.8%, Sepid Dane Com, Shiraz, Iran), various levels of yeast (1, 1.5, 2%w/w), and 65 ml of water. Then, dough was physically kneaded until it shaped a steady and elastic structure. Dough pieces were covered with a wet cloth, and fermentation was carried out at different temperatures (25, 30, and 35°C) for 120 min. Finally, the dough was divided into the equal parts of 50 g and the resultant pieces were baked in an oven at 250°C for 10 min.

### 2.4 Extraction of OTA

The extraction of OTA was carried out according to the method proposed by Institute of Standards and Industrial Research of Iran (ISIRI, 2011). Briefly, 4 g of samples (dough or bread) was transferred to a mixer. Then, 20 ml of methanol was added and agitated for 2 min. The obtained mixture was filtered through filter paper Whatman No. 1. 6‐mL aliquot was mixed with PBS to achieve a 40‐mL solution. Finally, 40 ml of the diluted solution was purified with a conditioned immunoaffinity column. The columns were then washed with 10 ml PBS at a flow rate of 1 ml/min and finally dried in an air stream for 2 min. OTA was eluted with methanol (0.5 ml) and poured into a vial and stored at −18°C until analysis. Finally, 100 µl of the elute was injected into the HPLC (ISIRI, 2011).

### 2.5 OTA analysis

The measurement of OTA was carried out by HPLC (Waters E2695; Waters Corporation, USA). The detection of OTA was done using 333 and 477 nm as wavelengths of excitation and emission, respectively. The separation was performed on Chromolith^®^ Performance RP‐18e (200 mm × 4.6 mm, i.d., 3 μm) with fluorescence detector and a particle size of 3 µ. The mobile phase delivered at a flow rate of 1 ml/min consisted of water: acetonitrile: methanol: acetic acid (30:39:30:1, v/v/v/v). HPLC measurements were carried out in triplicates.

### 2.6 Determination of pH and titratable acidity (TTA)

After the dough preparation, pH was measured with pH meter (PHS‐25, China). Titratable acidity was determined by the titration of 10 g sample by 0.1 N sodium hydroxide (NaOH). TTA is expressed in mL of 0.1 normal sodium hydroxide required to neutralize dough sample to a pH of 8.0 (Mantzourani et al., 2019).

### 2.7 Validation of the analytical method

The calibration curve, accuracy, the limit of detection (LOD), and the limit of quantification (LOQ) were determined to validate the used analytical method. The accuracy of OTA measurement method was checked by determining its recovery percentage in the spiking level of 5 and 2.5 µg/kg. Calibration curves (0.25–15 µg/ml) were drawn for OTA by analyzing the least‐squares linear regression of OTA peak area versus its concentration data. To calculate the detection limit (LOD) and the quantification limit (LOQ), three and ten times of the standard deviation of the blank sample to the slope of calibration line curve were used, respectively.

### 2.8 Statistical analysis

All statistical analyses were carried out using the Statistical Package for the Social Sciences (SPSS Inc.) version 16.0 for windows. One‐way analysis of variance (ANOVA) together with Duncan's method was applied to evaluate the variations in the levels of OTA. The correlation among OTA level, pH, and TTA was determined by Pearson coefficient. All processing and sampling steps were taken in triplicate.

## 3 RESULTS

### 3.1 Method validation

**Figure 1:**
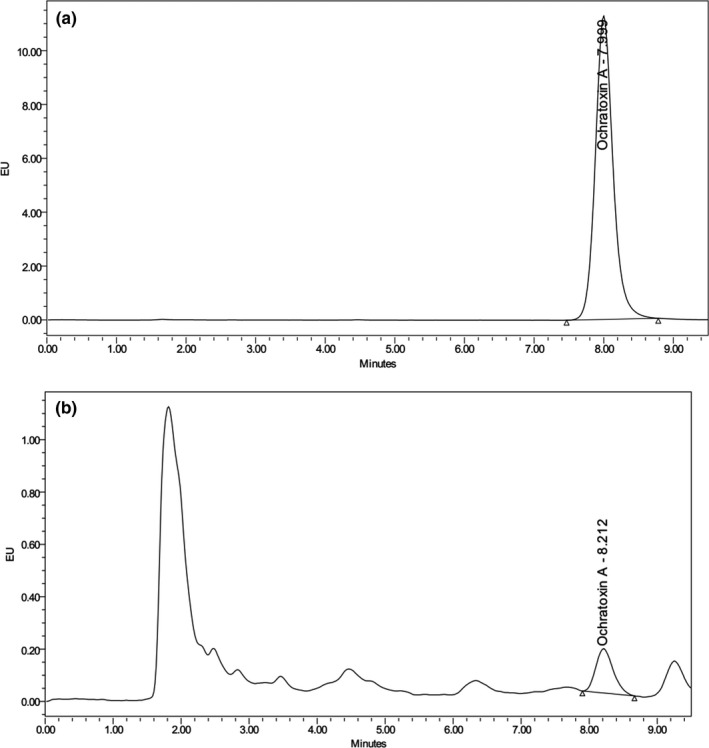
HPLC‐FLD chromatograms of (a) OTA standard solution (100 µg/L); (b) dough sample after fermentation

The chromatogram of OTA is shown in Figure 1 **.** For dough sample, the regression equation of calibration curve, *R* ^2^, LOD, and LOQ was *y* = 448096*x* + 424.71, 0.9985, 0.1 µg/kg, and 0.3 µg/kg, respectively. Validation data for flour and bread are shown in Table 1.

**Table 1:** Validation data for OTA (µg/kg) in flour, dough, and bread

Matrix type	Spiked concentration (µg/kg)	Mean recovery (%)	RSD (%)	LOD (µg/kg)	LOQ (µg/kg)
Flour	2.5	80.53	1.59	0.14	0.34
	5	81.67	1.66		
Dough	2.5	76.15	2.73	0.10	0.30
	5	74.90	1.64		
Bread	2.5	83.54	1.96	0.12	0.33
	5	86.24	2.62		

### 3.2 Effect of yeast level on OTA

According to Table 2, by increasing the amount of yeast from 1% to 2% w/w, the less amount of OTA remained in the dough sample. The lowest OTA change was observed in the dough prepared with 1% yeast, while the maximum effect was observed in sample containing 2% of yeast.

**Table 2:** The OTA content (µg/kg) in flour samples fermented by various yeast levels in different temperature

Yeast content (%)	Fermentation temperature (°C)		
	25	30	35
1	3.44 ± 0.19^Aa^	3.01 ± 0.05^Ba^	2.99 ± 0.27^Ba^
1.5	2.9 ± 0.04^Ab^	2.8 ± 0.26^Aa^	2.7 ± 0.09^Aa^
2	2.54 ± 0.12^Ac^	2.45 ± 0.21^Ab^	2.03 ± 0.34^Bb^

Note

Different superscript capital letters within a row indicate statistically significant differences (*p* < 0.05) among values. Different superscript small letters within a column indicate statistically significant differences (*p* < 0.05) among values.

### 3.3 Effect of fermentation temperature on OTA

As shown in Tables 2 and 3, with increasing the fermentation temperature from 25°C to 35°C, a greater level of OTA was lost. The lowest amount of OTA was witnessed at 35°C for three yeast levels. The maximum value of OTA observed at 25°C.

**Table 3:** Reduction content of OTA (%) during dough fermentation with various yeast levels in different temperature

Yeast content (%)	Fermentation temperature (°C)		
	25	30	35
1	31.17 ± 3.71^Bc^	39.81 ± 0.93^Ac^	40.28 ± 5.34^Ac^
1.5	41.97 ± 0.71^Ab^	43.98 ± 5.16^Ab^	45.99 ± 1.75^Ab^
2	49.23 ± 2.33^Ba^	51.08 ± 4.3^Ba^	59.41 ± 6.78^Aa^

Note

Different superscript capital letters within a row indicate statistically significant differences (*p* < 0.05) among values. Different superscript small letters within a column indicate statistically significant differences (*p* < 0.05) among values.

### 3.4 Relationship of pH and titratable acidity with OTA decrement

**Figure 2:**
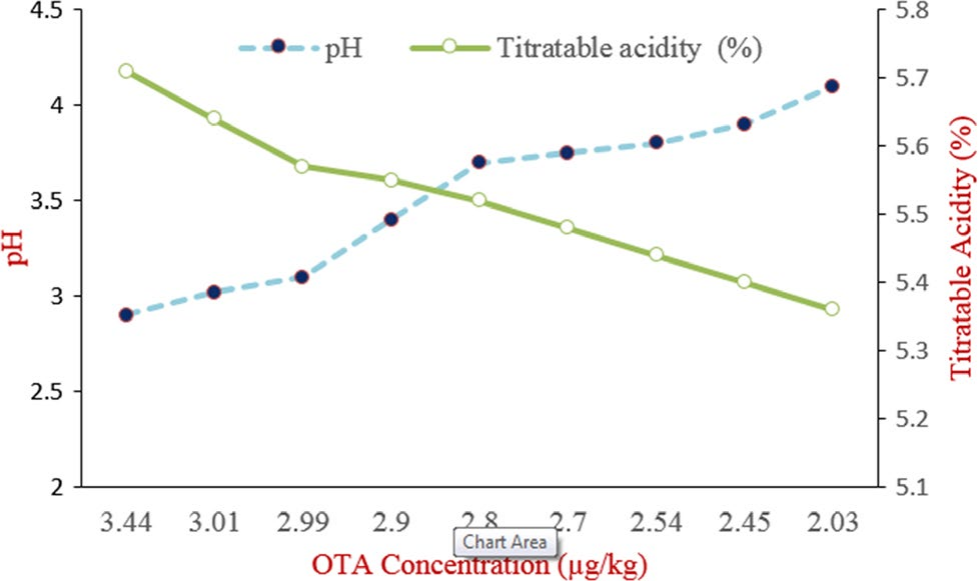
The relationship between OTA concentration changes during wheat flour fermentation with pH and acidity

As shown in Figure 2, the lowest concentration of OTA (2.03 µg/kg) in fermented dough was related to sample with the lowest pH (4.1) and the highest titratable acidity (5.36 ml). On the other hand, the decomposition of OTA was correlated with pH reduction (Pearson coefficient = 0.966, *p* < 0.001) and TTA increment (Pearson coefficient = 0.962, *p* < 0.001).

### 3.5 Effect of baking process on OTA

After selecting the optimal condition for the dough fermentation (2% yeast and fermentation temperature of 35°C), the samples of this treatment (containing OTA in level of 2.03 µg/kg) were placed in the oven for cooking. The OTA in baked sample was 1.64 µg/kg; therefore, it was reduced until 67.2%. During baking, OTA reduction was 19.21%.

## 4 DISCUSSION

Food safety is one of the major concerns of each society (Heshmati, 2015). The present study demonstrated the role of yeast level and the fermentation temperature on OTA reduction. In a previous study done by Milani & Heidari, 2017, the impact of active dry yeast, instant dry yeast, and compressed yeasts on OTA during wheat dough fermentation was investigated. The findings showed that compressed yeast had the strongest impact on the OTA during the bread fermentation process (Milani & Heidari, 2017). In current study, our finding showed that OTA reduction during fermentation depended on the compressed yeast and the fermentation temperature.

Our results on the OTA reduction during fermentation differed from the previous studies. In some of these studies, OTA was either not reduced, or its reduction was not significant, while in some other studies, it was declared that OTA content increased after fermentation (Vidal, Marín, et al., 2014; Vidal, Morales, et al., 2014). In a study done by Vidal, Morales, et al. (2014), sourdough addition into dough resulted in 29%–38% reduction of OTA; however, the statistical analysis showed these changes were not significant (Vidal, Marín, et al., 2014). The mean OTA content after kneading and the fermentation of flour containing high (9.5 ± 0.2 µg/kg), medium (5.1 ± 0.1 µg/kg), and low (0.8 ± 0.1 µg/kg) levels of OTA increased up to 19%; nonetheless, this increment was not statistically significant (Vidal, Morales, et al., 2014). Valle‐Algarra et al reported that 29.8% to 33.5% of OTA decreased during dough fermentation by compressed yeast at the temperature of 29–30ºC for 1 hr, which was lower than that obtained in the present study (Valle‐Algarra et al., 2009).

Our results regarding reduction of OTA during baking (19.21%) were higher than the findings of Vidal, Morales, et al. (2014). These authors reported that baking caused 9% reduction of OTA (Vidal, Marín, et al., 2014). However, Valle‐Algarra et al. (2009) reported more reduction 32.9) than our results for OTA during bread making (Valle‐Algarra et al., 2009).

In our study, OTA levels in flour and bread sample were 5 and 1.64 µg/kg, respectively; therefore, its reduction was 67.2%. However, these findings were not consistent with those of some previously published researches. Vidal, Morales, et al. (2014) declared that OTA level during bread making increased up to 24% (Vidal, Morales, et al., 2014). In other study, OTA level in flour sample was 8.6 and 0.8 µg/kg and its content reached to 9.9 and 1.1 µg/kg, showing the increment of 15.1%–37.5% (Vidal, Marín, et al., 2014).

This decrease in OTA level could be due to either the metabolites produced during the fermentation process or its binding to yeast wall. Carbon dioxide produced during the fermentation process reduced the pH of dough. In addition, alcohols and other compounds created by yeast could influence OTA (Milani & Heidari, 2017). Jayaram et al. (2013) recognized that succinic acid produced by the yeast itself, as one of the main pH‐determining metabolites, and carbon dioxide will only exert a minor impact on the dough pH. In the other words, the pH decreased in fermented dough was mainly caused by succinic acid production instead of carbon dioxide dissolution or bacterial organic acids (Jayaram et al., 2013).

The cause of greater reduction of OTA at higher fermentation temperatures could be attributed to more *S. cerevisiae* growth. There are several reports about the optimal growth temperature for yeast. Torija, Rozes, Poblet, Guillamón, and Mas (2003) studied the effects of temperature (15–35°C) on the growth of different strains of *S. cerevisiae* in white wine. Generally, the fermentation was faster at high temperatures (30–35°C) (Torija et al., 2003). Serra, Strehaiano, and Taillandier (2005) studied the influence of temperature and pH on the μ_max_ of *S. cerevisiae* growth. They disclosed that the temperature was also the factor with the principal effect on the response. The optimal temperature for *S. cerevisiae* at pH 5.0 was 35°C (Serra et al., 2005). Besides, Arroyo‐López, Orlić, Querol, and Barrio (2009) reported the highest μ_max_ value for *S. cerevisiae* was at 34.1°C (Arroyo‐López et al., 2009).

OTA decline mediated by microorganisms has been studied by many authors. It seemed that *S. cerevisiae* was capable of changing mycotoxins into less toxic substances based on the phenomenon of toxin binding to the cell wall, the production of degrading enzymes, and the action of biotransformation or biological transformation (Zhu, Hassan, Watts, & Zhou, 2016). Abrunhosa et al. (2014) assumed that the two main factors might be involved in the reduction, adsorption, and detoxification of OTA by various microorganisms. Primarily, OTA could be biodegraded through the hydrolysis of the amide bond linking the l‐β‐phenylalanine molecule to the OTα moiety. Subsequently, OTα and l‐β‐phenylalanine were almost nontoxic. Secondly, a more hypothetical process was involved in the degradation of OTA via the hydrolysis of the lactone ring (Abrunhosa et al., 2014).

Bejaoui, Mathieu, Taillandier, and Lebrihi (2004) investigated the ability of dead cells (treated with heat and acid) and live cells of *Saccharomyces* to remove OTA from the synthetic and natural grape juice. Their results indicated that the treatments of yeasts by heat and acidity significantly increased OTA removal compared with viable cells. They suggested that this phenomenon was due to the increased adsorption sites created by heat and acid treatments. Their results indicated that polysaccharides and peptidoglycans were treated with heat and acid. The heat caused protein denaturation or the formation of the Millard reaction products. Acid conditions affected polysaccharides by releasing monomers and creating aldehydes after breaking glucose bonding. These released products provided more adsorption sites than live cells and could increase surfaces for OTA binding (Bejaoui et al., 2004). Ringot et al. (2005) surveyed OTA adsorption by three types of yeast industry products: a vinasse containing yeast cell walls (EX16), a purified yeast beta glucan (BETA), and a yeast cell wall fraction (LEC). Results showed that yeast cell wall fraction was an effective adsorbent for OA removal (Ringot et al., 2005). In addition to absorption phenomenon between OTA and yeast cells, Cecchini, Morassut, Moruno, and Stefano (2006) reported that there was an independent interaction between OTA and phenolic compounds or other metabolic compounds produced by yeast. These metabolites, possibly linked to phenolic compounds, increased the surface for OTA binding (Cecchini et al., 2006). *Saccharomyces* and non*‐Saccharomyces* yeasts had the capability of reducing 0.6 to 42.8% OTA. Some of the important factors affecting this phenomenon consisted of the test conditions (testing on a laboratory scale or living scale), strain, cell size, and toxin concentration (Petruzzi et al., 2014).

Joannis‐Cassan, Tozlovanu, Hadjeba‐Medjdoub, Ballet, and Pfohl‐Leszkowicz (2011) investigated the efficiency of yeast‐based products for the absorption of the three mycotoxins: zearalenone, AFB1, and OTA. Eight products (yeast cell wall or inactivated yeast) were tested. The results showed that the best product, a yeast cell wall from baker's yeast, had the ability to absorb 68% ZEA, 29% AFB1, and 62% OTA. The absorption phenomenon mainly depended on both composition yeast and mycotoxins. They believed that the adsorption mechanisms were complex for the yeast products, perhaps since more than one site was accessible for mycotoxin, while the configuration and shape of the sorption site were important (Joannis‐Cassan et al., 2011).

The reason for the difference between our results and previous studies, regarding the effect of fermentation on the OTA reduction, could be due to differences in the OTA concentration, fermentation conditions such as period and temperature, pH, and dough combination containing microorganisms and enzymes (Schaarschmidt & Fauhl‐Hassek, 2018).

Bread baking might cause degradation of OTA, which mostly depended on the thermostability of OTA, pH level, and the baking time. The use of yeast or sourdough bread, probably led to the activation of enzymes by yeast, causing the destruction, loss of OTA or its sensitivity to heat (Mansouri‐Nasrabadi et al., 2018). Therefore, OTA could be eliminated or reduced during the bread baking despite being resistant to heat.

## 5 CONCLUSION

In general, our results revealed that the content of by compress yeast of *Saccharomyces* and fermentation temperature had significant impact on OTA during bread fermentation. Increasing the amount of *S. cerevisiae* from 1% to 2% and the fermentation temperature from 25 ºC to 30 ºC resulted in the increment of OTA reduction from 31.17% to 59.41%. During baking, OTA reduction was 19.21%. In general, the utilization of 2% w/w *S. cerevisiae* and the fermentation temperature of 35 ºC could reduce considerable amount of OTA in wheat bread. Finally, it can be concluded that the yeast of *S. cerevisiae* (commonly known as baker's yeast) had the ability to eliminate OTA with the lowest cost and high efficiency.

## CONFLICT OF INTEREST

The authors declare that they do not have any conflict of interests.

## ETHICAL APPROVAL

This study was approved by Sari Agricultural Sciences and Natural Resources University. This study does not involve any human or animal testing.

## INFORMED CONSENT

None.
